# DNA-Dependent Protein Kinase Is a Context Dependent Regulator of Lmx1a and Midbrain Specification

**DOI:** 10.1371/journal.pone.0078759

**Published:** 2013-10-23

**Authors:** Cameron P. Hunt, Stewart A. Fabb, Colin W. Pouton, John M. Haynes

**Affiliations:** Drug Discovery Biology, Monash Institute of Pharmaceutical Sciences, Monash University (Parkville), Melbourne, Australia; Rutgers University, United States of America

## Abstract

The identification of small molecules capable of directing pluripotent cell differentiation towards specific lineages is highly desirable to both reduce cost, and increase efficiency. Within neural progenitors, LIM homeobox transcription factor 1 alpha (Lmx1a) is required for proper development of roof plate and cortical hem structures of the forebrain, as well as the development of floor plate and midbrain dopaminergic neurons. In this study we generated homologous recombinant cell lines expressing either luciferase or β-lactamase under the control of the *Lmx1a* promoter, and used these cell lines to investigate kinase-mediated regulation of Lmx1a activity during neuronal differentiation. A screen of 143 small molecule tyrosine kinase inhibitors yielded 16 compounds that positively or negatively modulated Lmx1a activity. Inhibition of EGF, VEGF and DNA-dependent protein kinase (DNA-PK) signaling significantly upregulated Lmx1a activity whereas MEK inhibition strongly downregulated its activity. Quantitative FACS analysis revealed that the DNA-PK inhibitor significantly increased the number of Lmx1a+ progenitors while subsequent qPCR showed an upregulation of Notch effectors, the basic helix-loop-helix genes, *Hes5* and *Hey1*. FACS further revealed that DNA-PK-mediated regulation of Lmx1a+ cells is dependent on the rapamycin-sensitive complex, mTORC1. Interestingly, this DNA-PK inhibitor effect was preserved in a co-culture differentiation protocol. Terminal differentiation assays showed that DNA-PK inhibition shifted development of neurons from forebrain toward midbrain character as assessed by Pitx3/TH immunolabeling and corresponding upregulation of midbrain (*En1*), but not forebrain (*FoxG1*) transcripts. These studies show that Lmx1a signaling in mouse embryonic stem cells contributes to a molecular cascade establishing neuronal specification. The data presented here identifies a novel regulatory pathway where signaling from DNA-PK appears to suppress midbrain-specific Lmx1a expression.

## Introduction

Small molecule-directed differentiation of mouse embryonic stem cells (mESCs) toward distinct neural subtypes is an attractive proposition that will facilitate the large-scale generation of subtype specific neurons for use in drug screening assays and cell replacement therapies. The LIM homeobox transcription factor 1α, Lmx1a, is expressed by neural progenitors within the ventral midbrain, notochord, roof plate and the otic vessels [1,2]. It is a major mediator of dopaminergic development where it cooperates with other signaling proteins; Wnt1, Shh and FoxA2 [3–7] to regulate dopaminergic fate. To this end, enriching for dopaminergic progenitors during differentiation of mESCs by forced expression of Lmx1a has shown promise [8]. However, our previous work has established that during neural induction, in the absence of specific cues for floor plate development, most of the Lmx1a positive neurons commit to a forebrain phenotype [2]. Generally, specific temporal cues are required to induce midbrain dopaminergic development [9] and under well-defined and appropriate differentiation paradigms, Lmx1a is an important mediator of dopaminergic development. 

At later stages of maturation, other markers of midbrain identity are often used to assess dopaminergic differentiation; these include the paired-like homeodomain transcription factor 3 (Pitx3) and/or tyrosine hydroxylase (TH) [10]. Pitx3 expression is initiated by immature midbrain neurons and is essential for their proper differentiation and survival [11,12] with fully differentiated adult midbrain dopaminergic (mDA) neurons generally positive for both TH and Pitx3 [13]. Attempts to generate mature dopaminergic neurons *in vitro* rely heavily on the use of recombinant proteins acting as growth factors or morphogens to modulate specific pathways [14,15]. These proteins are, however, expensive and can have limited effectiveness in directing ESC development due to batch-to-batch variability. These limitations of polypeptide growth factors have stimulated investigations of small molecule-dependent differentiation paradigms based on modulators of known signaling pathways [16,17]. In this study, we employed a small molecule screening strategy using protein kinase inhibitors to identify novel signaling pathways that may contribute to dopaminergic neurogenesis. We initially screened for molecules that were able to upregulate Lmx1a activity, and subsequently investigated the influence of small molecules in more detail by tracking the fate of neuronal progenitors as they became post-mitotic. 

## Materials and Methods

### Generation of reporter lines

The genetic reporter cell lines used in this study included *Lmx1a-luc-IRES-eGFP and Lmx1a*-*AMP-IRES-eGFP* mESCs. Vectors were designed to replace exon 1 of one allele of the *Lmx1a* gene with cDNA encoding for either firefly luciferase + eGFP or β-lactamase + eGFP, the two pairs of cDNA were separated by an internal ribosome entry site (IRES) in each case (i.e. β-lactamase*-IRES-eGFP*) . We also used a *Pitx3-eGFP* reporter cell line also derived from E14Tg2a cells and previously described [18]. See Figure **S3** for further details of the targeting vectors.

### Neural induction and differentiation

E14Tg2a mouse ESCs (ATCC, USA), and genetic reporter cell lines were maintained in mESC medium of DMEM containing GlutaMAX™-I supplemented with 10% (v/v) FCS (ES qualified), 100 units/mL Penicillin/Streptomycin, 0.1 mM β-mercaptoethanol (all from Life Technologies, Australia) and 10^3^ units/mL Leukemia inhibitory factor (LIF, Merck Millipore, Australia). Cells were passaged on 0.1% (v/v) gelatin-coated culture plates every other day. 

### Generation of neural progenitors

Neural differentiation was achieved as described previously [19] using serum-free N2B27 medium to induce neural differentiation. N2B27 is a 1:1 mixture of modified Neurobasal® and modified DMEM/F-12. Modified Neurobasal consists of Neurobasal® medium and 1x serum-free B27 supplements (both Life Technologies, Australia). Modified DMEM/F-12 consists of DMEM/F-12 medium, 1x N2 supplement, 0.005% (v/v) Fraction V BSA (all Life Technologies, Australia) and 1 mg/mL Bovine insulin (Gemini Bio-products, USA). Briefly, mESCs were seeded at 5 x 10^3^ cells/cm^2^ in complete mESC medium, as described above. Around 48 hours later, cells were washed with 1x PBS and incubated in serum-free N2B27 medium to induce neural differentiation (day 0). Cells were differentiated in N2B27 with medium replaced every other day until day 8, where Lmx1a expression appears to plateau [2].

### Small molecule tyrosine kinase inhibitor libraries

The majority of small molecule compounds screened were from two commercially available kinase inhibitor libraries (Cat # 539744 and #539745, Calbiochem, USA). Compounds were screened at a concentration ten times higher than the reported IC_50_ concentration and stored according to manufacturer’s specifications. A total of 143 inhibitors were screened using 96-well format from a possible 160 in the Calbiochem libraries. The remaining inhibitors were not supplied in sufficient mass to allow for screening at 10 x IC_50_. Other small molecule signaling pathway inhibitors used included: LY294002 (PI3K inhibitor; 14 µM, Cell Signaling Technologies, USA), VO-OHpic trihydrate (PTEN inhibitor; 1.25 and 3.5 µM, Sigma-Aldrich, USA), Akt inhibitor VIII (0.58 µM, Calbiochem, USA) and U-73122 (PLC-γ inhibitor; 3.0 mM Cayman Chemicals, USA). All compounds were dissolved in DMSO (with the exception of VO-OHpic hydrate which was dissolved in 1:1 DMSO: H_2_O). The final concentration of DMSO used in the cell culture medium was 0.5% (v/v). At least three independent experiments were performed using quadruplicate wells in each case.

### Cell viability and luciferase assays for small molecule screening

Neural progenitors (i.e. mESCs undergoing differentiation) were treated with small molecules at day 4 and 6 of differentiation in fresh N2B27 containing 0.5% (v/v) DMSO or small molecules in the presence of the same concentration of DMSO. On day 8 of differentiation, CellTiter-Blue® (CTB) reagent (Promega, USA) was added to the culture plate for 4 hours at 37°C. The plates were then analysed using the EnVision® 2101 Multilabel plate reader (PerkinElmer, Australia) to assay the metabolized product at 590 nm. After performing the viability assay, the CTB solution was washed out with 1x PBS and replaced with Steady-Glo® luciferase substrate (Promega, USA), plates were then incubated in the dark at RT for 10 min. Luminescence was quantified using the EnVision® plate reader. 

### Flow cytometry

Single-cell suspensions were prepared by enzymatic digestion (Accutase® Invitrogen, USA) of adherent cells. The *Lmx1a*-*AMP-IRES-eGFP* cell line was assayed for β-lactamase expression using the Live BLAzer™ FRET-B/G loading kit (Invitrogen, USA). Briefly, cells were incubated with loading dye in the dark at RT for 2.5-3 hours. After incubation, cells were centrifuged at 200xg and resuspended in FACS buffer (10% (v/v) FCS in 1x PBS) containing the cell viability dye Sytox Blue® at 5 µM (Invitrogen, USA). Quantitation and/or sorting of Lmx1a+ cells was performed on either FACS Canto II (BD, Australia) or MoFlo® Astrios (Beckmann Coulter, USA) cytometers. 

### Western Blotting

Protein lysates were isolated from confluent 6-well plates in ice-cold RadioImmunoPrecipitation Assay (RIPA) buffer containing protease and HALT™ phosphatase inhibitor cocktails (Roche, USA and Thermo Scientific, Australia, respectively). Protein quantitation was performed using DC Protein Assay kit (Bio-Rad, Australia). Samples were stored at -80°C until required. On the day of use, mammalian target of rapamycin (mTOR) and β-actin proteins were separated by SDS-PAGE at 100V for 90 min using 6% and 15% acrylamide gels, respectively. Proteins were blotted onto Nitrocellulose membranes (GE, Australia) using the Trans-Blot SD Semi-Dry Transfer Cell (Bio-Rad Laboratories, USA) at 20 V for 2 hours at 4°C. Membranes were probed for native mTOR and phospho-mTOR (Ser2448) (Cell Signaling Technologies, USA), with β-actin (Santa-Cruz, USA) used as a loading control. 

### Further differentiation of neural progenitors

At day 10 of differentiation, 7 x 10^4^ cells/cm^2^ were subcultured and replated into laminin-coated (2 µg/cm^2^) culture wells, where, N2B27 medium was supplemented with morphogens; Fibroblast growth factor 2/basic (Fgf2; 10 ng/mL, R&D Systems, Australia) and Heparin Sulphate (HS; 10 ng/mL, Sigma-Aldrich, Australia). Medium was replaced with fresh N2B27 without morphogens 24 hours after replating. On day 14, N2B27 was supplemented with glial-derived neutrophic factor (GDNF; 10 ng/mL, R&D Systems, Australia) and ascorbic acid (AA; 200 µM, Sigma, Australia). Cells were maintained in this medium until analysis with fresh medium added every other day.

### Immunocytochemistry and cell counting

#### Immunocytochemistry

Cells were fixed in 4% (w/v) paraformaldehyde, permeablized using 1x PBS with 0.1% (v/v) Triton™ X-100, blocked in 2% (v/v) donkey serum and incubated with primary antibodies overnight at 4°C. Following incubation, cells were labeled with donkey anti-rabbit Alexa Fluor® 594 and donkey anti-mouse Alexa Fluor® 488 (both from Life technologies, Australia) secondary antibodies. Cells were counterstained with either TO-PRO®-3 or SYTO® blue DNA probes (Life technologies, Australia) and imaged using Nikon A1 confocal microscope (Nikon, Japan). See Table **S1** for full list of antibodies used. 

#### Cell counting

To assess total numbers of cells positive for a particular marker, images from nine random fields-of-view (FOV) were taken in each well of a 24-well plate. Cells were counterstained with either TO-PRO®-3 or SYTO® fluorescent probes. Total cell numbers per FOV were then quantified using the thresholding and automatic cell counting tools found in the Nikon elements software. These numbers were used to estimate total cells positive for post-mitotic markers across the nine FOVs. 

### Isolation of mRNA and qPCR analysis

Total RNA was extracted from cells using either High Pure RNA isolation Kit (Roche, Australia) or RNeasy™ Micro kit (Qiagen, Australia). RNA was quantified using a NanoDrop® 1000 benchtop spectrophotometer (Thermo Scientific, USA) and converted to cDNA using the First stand cDNA synthesis kit (Roche, Australia). For qPCR, 3 ng of cDNA was used for analysis with a CFX96™ Real-time cycler (Bio-Rad, Australia). Primer sequences used for PCR experiments are detailed in Table **S2**. 

### Statistical analysis

Results from experiments are presented as mean ± standard error of the mean (SEM) of at least 3 replicate experiments. Statistical analysis was performed on raw data by one-way analysis of variance (ANOVA) with post-hoc Dunnett’s or Bonferroni’s test using PRISM v6.00 (GraphPad Software, USA). In all cases, a p-value of 0.05 was considered significant. Real-time qPCR analyses were performed using the 2^-ΔΔCt^ method originally described elsewhere [20].

## Results

### Validation of reporter lines

Knock-in reporter mESC lines were generated by electroporation of targeting vectors (Figure **S3**) into wild-type (E14Tg2a) mESCs. Full details validating the *Lmx1a*-*AMP*-*IRES*-*eGFP* mESC line have been described previously [2,21]. In the case of the *luc*-*IRES*-*eGFP*-containing construct, successful integration into the *Lmx1a* locus was initially confirmed by PCR using genomic DNA from surviving mESC colonies and primers for genomic *Lmx1a*. Southern Blotting was subsequently used to confirm correct integration by hybridisation of probes complementary to the *Lmx1a* sequence upstream of the 5’ homology arm of the vector and downstream of the 3’ homology arm, Figure **S3C**. 

### Modulation of Lmx1a reporter activity by small molecules

**Figure 1:**
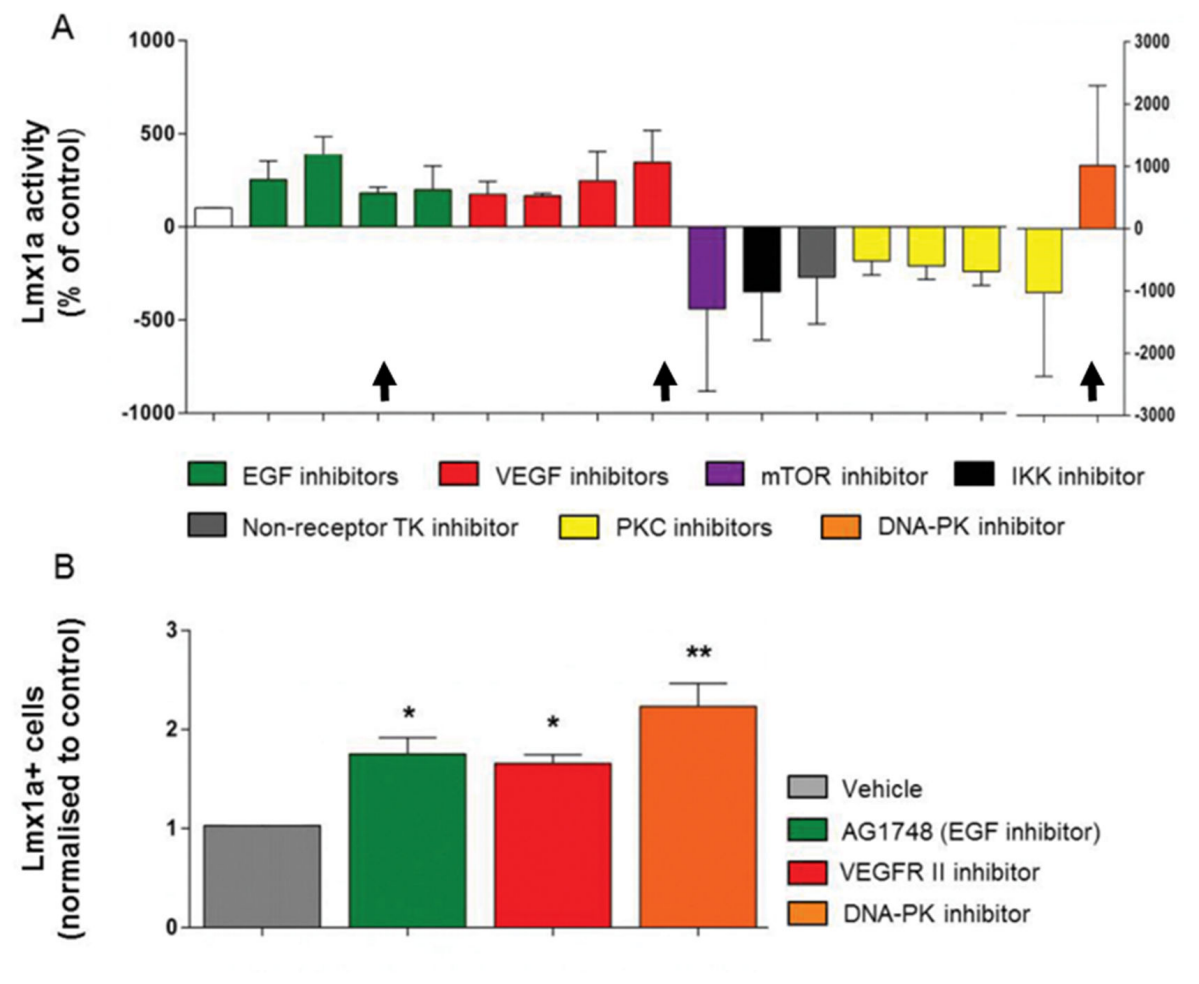
Small molecule inhibitor screens reveal multiple inhibitor classes effect Lmx1a reporter gene expression. 143 kinase inhibitors were screened yielding 16 small molecule agents (SMAs) for further analysis. Panel A shows Luciferase activity on day 8 of differentiation after incubation of *Lmx1a*-*luc* mESCs with SMAs from day 4 to day 8. Panel B shows that incubation with inhibitors that promote Lmx1a activity also increases the generation of Lmx1a+ cells. Color coded bars in A represent different SMAs with known similar kinase inhibitory activity, where black arrows indicate SMAs selected for analysis in panel B See Figures **S1** and **S2** for compound libraries used. Data expressed as mean ± SEM of at least four independent experiments. **p<0.01 and *p<0.05 one-way ANOVA with Bonferroni’s post-hoc test, n=4.

**Figure 2:**
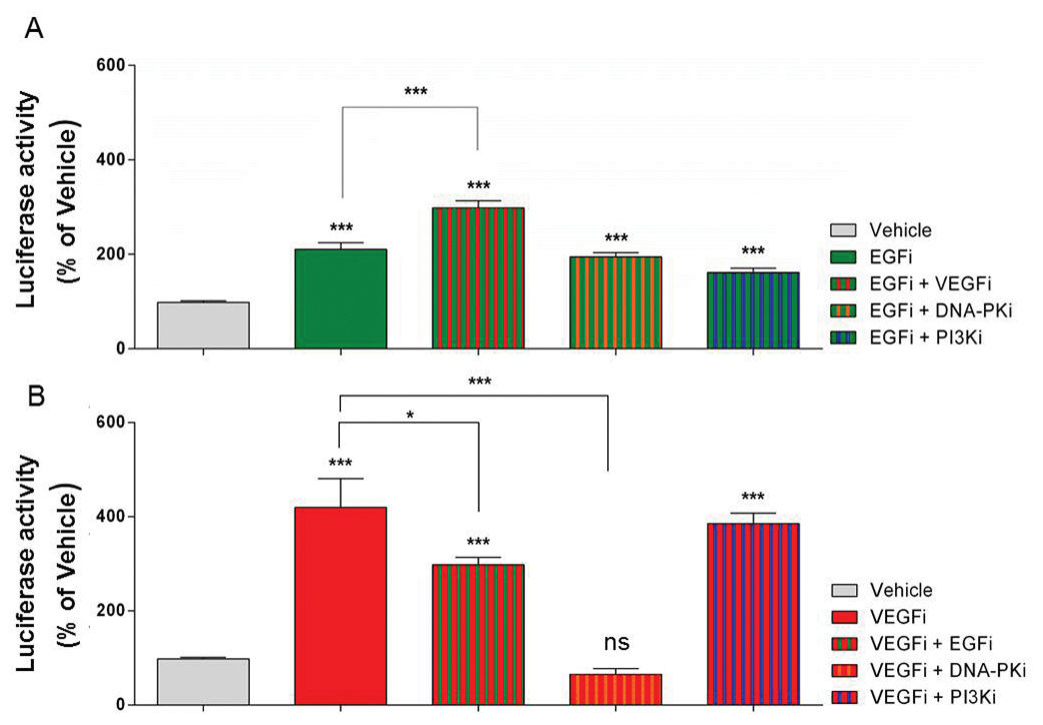
Modulation of Lmx1a reporter gene expression by combinations of small molecule kinase inhibitors. Luciferase activity was assayed on day 8 after exposure of differentiating mESCs to combinations of SMAs from day 4-8. (A) Activity of EGF inhibitor plus other SMAs (B) Activity of VEGF inhibitor plus other SMAs, ***p<0.001 and *p<0.05, one-way ANOVA using Bonferroni’s multiple comparison test. Data expressed as mean ± SEM, n=4.

We performed plate-based screening assays for luciferase activity using *Lmx1a-Luc-IRES-eGFP* mESCs after incubation with kinase inhibitors. We subsequently identified 16 small molecule agents (SMAs) that regularly altered luciferase expression by day 8. SMAs that consistently increased luciferase activity around 2-fold, relative to vehicle controls, included inhibitors of vascular endothelial growth factor (VEGF), epidermal growth factor (EGF) and DNA-dependent protein kinase (DNA-PK) pathways. SMAs that inhibited Iκβ kinase (IKK), mTOR, and Protein Kinase C (PKC) signaling pathways decreased luciferase approximately 3-fold below vehicle control (Figure **1A**). Importantly, these compounds altered luciferase activity without significantly affecting total cell viability (Figure **S1**). One SMA from each inhibitor class that increased luciferase activity was selected for further analysis using FACS to determine if luciferase activity correlated with the generation of Lmx1a+ cells. In the cases of EGF and VEGF inhibitors, where multiple small molecules could be chosen, compounds were selected based on potency and or variability of luciferase activity. To verify whether the effects of SMAs were additive, combinations of kinase inhibitors were added to cultures. Compared to the inhibition of EGF alone, the addition of a VEGF inhibitor increased luciferase activity (p<0.01, one-way ANOVA with post-hoc Bonferroni’s test, n = 4, Figure **2A**). Phosphoinositide 3-kinase (PI3K) signaling is a key component of receptor tyrosine kinase (RTK) signal transduction. To investigate the possibility that the luciferase activity observed in the absence of EGF signaling was still RTK/PI3K-dependent, we incubated cells with the PI3K inhibitor, LY294002. No further change in luciferase activity was seen in these culture wells (Figure **2A**); moreover, the addition of the DNA-PK inhibitor, AMA-37, did not modify the elevation of luciferase activity seen following EGF inhibition, Figure **2A**. 

**Figure 3:**
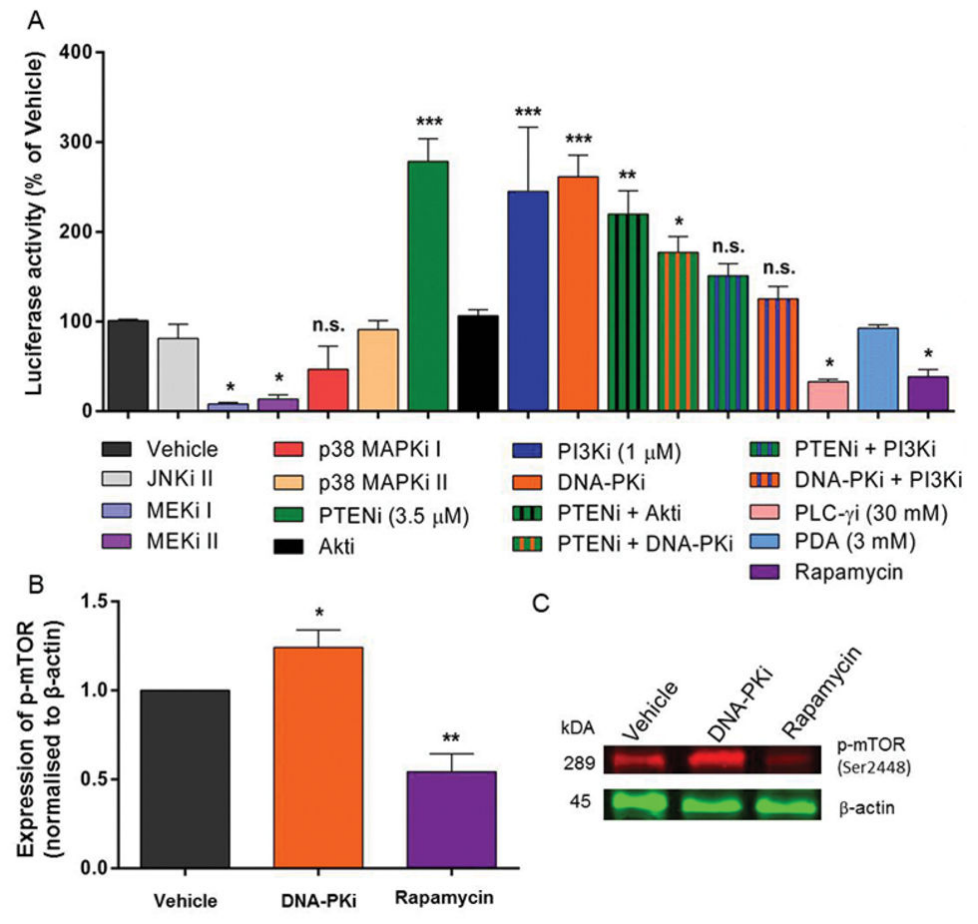
Activation of PI3K and PIKK signaling pathways induces Lmx1a reporter gene expression. Panel A shows the luciferase activity from *Lmx1a*-*luc* mESCs on day 8, after incubation with combinations of SMAs from day 4-8. PD169316 and SB220025 are inhibitors of p38 MAPK pathway whereas U-73122 specifically inhibits PLC-γ signaling. Solid colored columns represent incubations with single SMAs whereas striped columns depict combination incubation of SMAs. Western Blot analysis (B and C) revealed that DNA-PKi induces phosphorylation of mTOR, another member of the PIKK superfamily, whilst Rapamycin inhibited its phosphorylation. Membranes were probed for mTOR specifically phosphorylated at Serine 2448 (Ser2448) and β-actin using primary antibodies. Proteins were visualized using donkey anti-rabbit AlexaFluor 680 and donkey anti-mouse IE-800 for mTOR and β-actin, respectively. Data presented as mean ± SEM, ***p<0.001, **p<0.01 and *p<0.05 for one-way ANOVA compared to vehicle using Bonferroni’s post-hoc test, n=3. Abbreviations: PTENi- Phosphatase tensin homologue inhibitor, JNK- Janus Kinase, MEK- Mitogen-activated protein kinase kinase, PDA- Phorbol di-acetate and mTOR- Mammalian target of rapamycin.

Compared to VEGF inhibitor alone, the addition of the EGF inhibitor (EGFi + VEGFi), reduced luciferase activity (p<0.05, one-way ANOVA, post-hoc Bonferroni’s test, n = 3) while the addition of DNA-PK inhibitor (VEGFi + DNA-PKi) completely abolished this increase, reducing Lmx1a activity (p<0.001, one-way ANOVA, post-hoc Bonferroni’s test, n = 3) to control levels (Figure **2B**). The inhibition of both PI3K and VEGF signaling had no additional effect on luciferase activity (Figure **2B**), whilst incubation with DNA-PKi increased luciferase activity (p<0.001, one-way ANOVA, post-hoc Bonferroni’s test, n = 3, Figure **3A**).

### Intracellular signaling pathways that modulate Lmx1a reporter gene expression

To further investigate which signaling pathways were responsible for regulating *Lmx1a* promoter activity, neural progenitors were exposed to a series of compounds that modulated mitogen-activated protein kinase (MAPK), c-Jun N-terminal kinase (JNK), phospholipase C (PLC), and PI3K-related signaling pathways, some in combination with the inhibitor of DNA-PK (Figure **3A**). The p38 MAPK inhibitors SB220025 and PD169316, JNK inhibitor (JNK inhibitor II), the PKC agonist Phorbol di-acetate (PDA) and the Akt inhibitor had no significant effect on expression of Lmx1a (one-way ANOVA, post-hoc Bonferroni’s test, n=3). Incubation with inhibitors of phosphatase tensin and homolog (PTEN), PI3K and DNA-PK, as expected from Figure **1**, were able to significantly (p<0.05) promote reporter activity by day 8. Combinations of PTEN + PI3K and DNA-PK + PI3K had no significant effect (p>0.05) on luciferase expression whereas PTEN inhibition in combination with either inhibition of Akt, or DNA-PK signaling, were able to significantly (p<0.05) promote luciferase activity by day 8 (Figure **3A**). In contrast, incubation with MAPK kinase (MEK1 and MEK2) inhibitors and the PLC-γ inhibitor (U-73122) caused a significant (p<0.05) decrease in reporter activity (Figure **3A**). Viability of cells incubated with inhibitors from Figure **3A** is shown in Figure **S2** **.**

To confirm that incubation with DNA-PKi did not inhibit other proteins within the PI3K-related kinase (PIKK) family; such as mammalian target of rapamycin (mTOR), total protein lysates were separated by electrophoresis and analysed by Western blot. These data show that incubation with DNA-PKi does not inhibit mTOR signaling and that phosphorylation of mTOR (Ser2448) is increased in response to inhibition of DNA-PK, Figures **3B and C**.

### Quantitative Lmx1a expression by FACS

**Figure 4:**
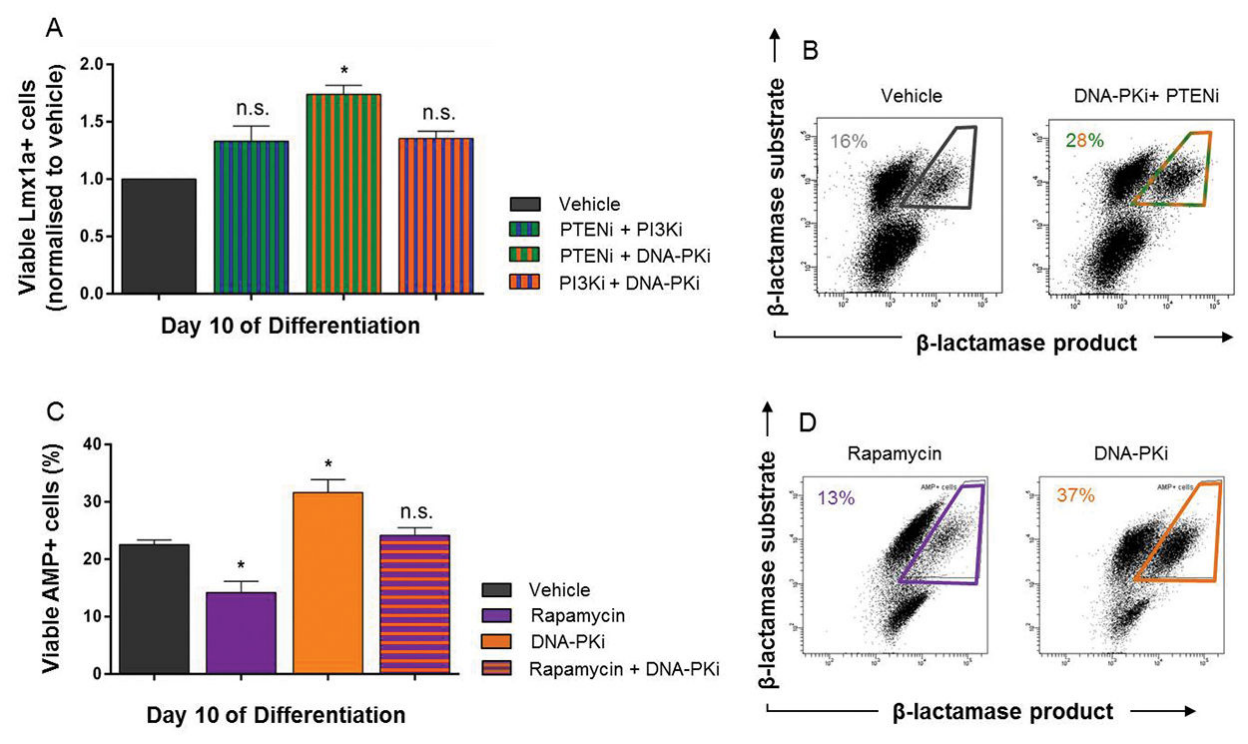
The effect of PI3K and PIKK signaling on Lmx1a+ cells. The involvement of PI3K, DNA-PK and mTOR signaling on the generation of Lmx1a+ cells using FACS is shown in panels A-D. Bar graphs (A and C) were quantified from dot plots, representative plots are shown in panels B and D. During combination experiments with modulators of PI3K and DNA-PK, large variability in the number of Lmx1a+ cells were observed, therefore, data for these experiments is expressed as fold difference from vehicle control. Column graphs expressed as mean ± SEM of at least three independent experiments. ***p<0.001, **p<0.01 and *p<0.05, one-way ANOVA with Bonferroni’s post-hoc test, n=3.

Control over PIKK signaling pathways by PI3K and DNA-PK appears essential in regulating Lmx1a activity. To further explore the involvement of these signals, *Lmx1a*-*AMP* mESCs were incubated with combinations of DNA-PK, PI3K, PTEN and mTOR inhibitors and analysed by FACS, Figure 4A-D. Flow cytometry revealed that inhibition of PTEN and DNA-PK significantly (p<0.05, one-way ANOVA, post-hoc Bonferroni’s test, n=3) increased the number of AMP+ cells by day 10 to 1.73 ± 0.11-fold compared to vehicle (Figures **4A and B**), whereas, inhibition of PI3K with either DNA-PKi or PTENi had no significant effect 1.35 ± 0.18 and 1.33 ± 0.22-fold, respectively Figure **4A**. Quantitative FACS analysis also revealed that although combination of Rapamycin + DNA-PK inhibitors did not significantly (p>0.05) effect generation of Lmx1a+ cells (Figure **4C**), incubation with Rapamycin and DNA-PKi alone were able to significantly (p<0.05) decrease and increase the percentage of Lmx1a+ cells to 13.0 ± 1.8% and 37.0 ± 2.1% compared to vehicle (Figures **4C and D**), respectively. 

### Downstream targets of DNA-PK signaling

**Figure 5:**
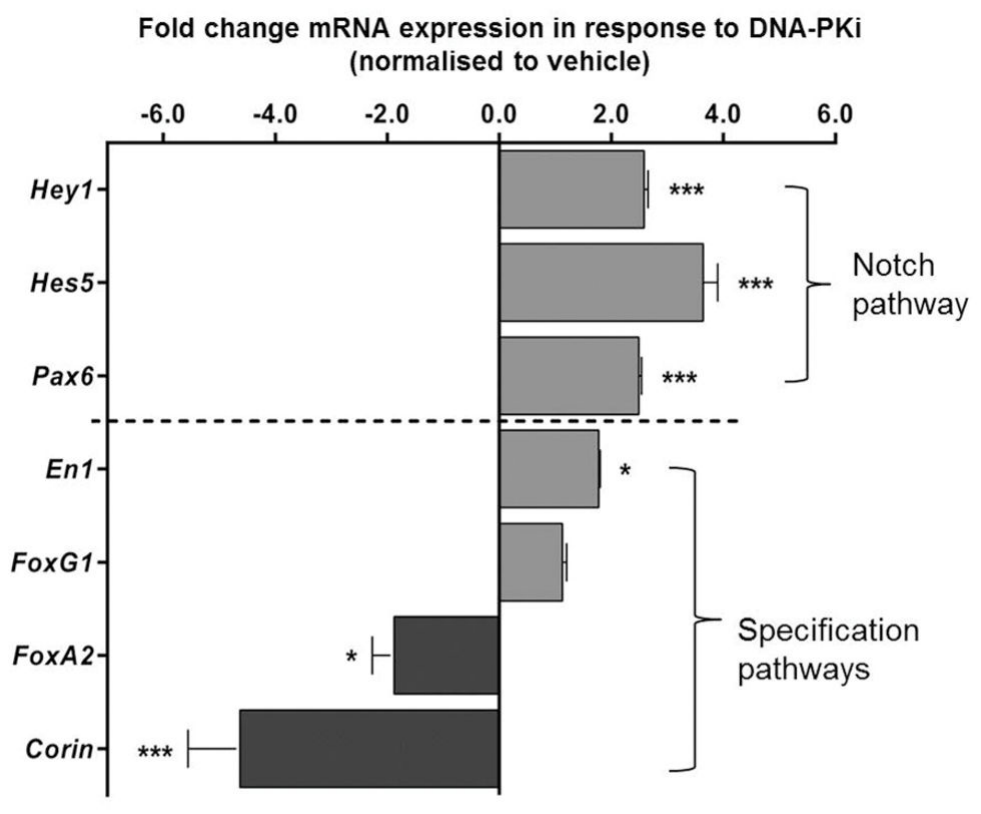
DNA-PK inhibition promotes regional specification and upregulation of Notch pathway transcripts. Real-time PCR analyses for transcripts of Notch signaling and regional specification using Lmx1a+ progenitors after isolation by flow cytometry. Cells exposed to DNA-PKi for four days (days 4-8), significantly (p<0.01) upregulated the Notch effector genes *Hes5* and *Hey1* as well as the Notch target, and RG marker *Pax6*, by day 8. Incubation with DNA-PKi during this period also upregulated expression of the midbrain marker Engrailed 1 (En1), however, expression of forebrain marker (FoxG1), also known as brain factor 1 (BF-1), was unchanged in these cultures. DNA-PKi-incubated cells also significantly downregulated the expression of *FoxA2* and *Corin*, two markers that demarcate progenitors of the ventral neural tube. Data presented as mean ± SEM of at least four independent experiments ***p<0.001, **p<0.01 and *p<0.05, respectively, using one-way ANOVA with Bonferroni’s post-hoc test, n=3.

Although there is little evidence for DNA-PK controlling any aspect of dopaminergic development, signaling from PI3K and PIKK superfamily members has been reported to cross-talk with the Notch signaling pathway to regulate dopaminergic neurogenesis [22]. Therefore, we investigated downstream genetic regulation of the Notch pathway in response to DNA-PK inhibition. During differentiation, *Lmx1a-AMP-IRES-eGFP* progenitors were sorted based on β-lactamase activity by FACS and analysed for expression of Notch effector genes using qPCR. Inhibition of DNA-PK significantly (p<0.05 one-way ANOVA post-hoc Bonferroni’s post-test n=4) upregulated transcription of *Hairy and enhancer of Split 5 (Hes5*) by 3.5 ± 0.8-fold and *Hairy/enhancer-of-split related with YRPW motif protein* 1 (*Hey1*) by 2.8 ± 0.2-fold compared to vehicle in Lmx1a+ cells, Figure **5**. Moreover, Figure **5** shows that transcription of *Pax6*, a major signaling target of Notch, was significantly upregulated (2.4- ± 0.2-fold, p<0.05) in response to DNA-PKi. The Lmx1a+ fraction isolated by FACS also revealed a significant (p<0.05, 1.9 ± 0.1-fold) increase in the expression of mRNA for the midbrain marker *Engrailed 1* (*En1*), but not (1.02 ± 0.1-fold, relative to controls) the forebrain marker *Forkhead box G1* (*FoxG1*). Lastly, incubation with DNA-PKi also significantly down regulated expression of ventral midbrain markers, *Forkhead box protein A2* (*FoxA2*) and *Corin* to 1.8 ± 0.4 and 4.6 ± 1.0-fold of vehicle, respectively.

### Investigating the influence of small molecules during co-culture differentiation

**Figure 6:**
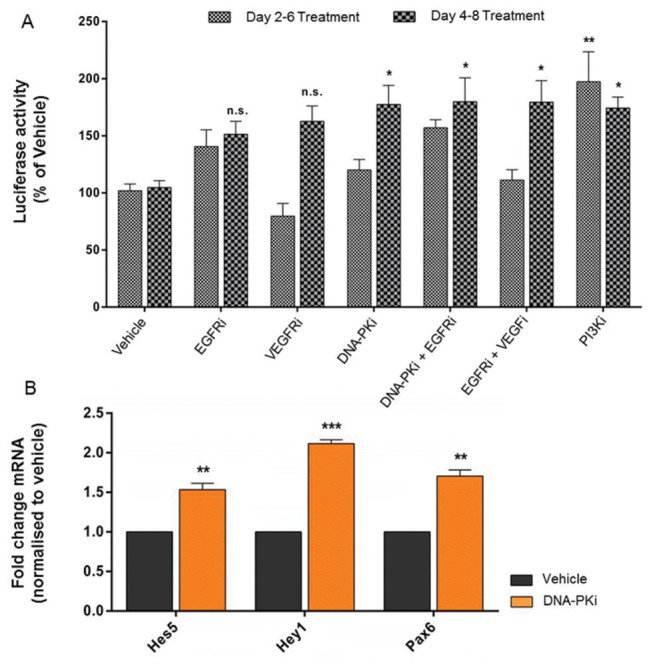
Small molecules can regulate Lmx1a activity and the Notch pathway after PA6-mediated neural induction. Panel A shows that *Lmx1a*-*luc* mESCs differentiated by co-culturing with PA6 stromal cells and incubated with small molecule inhibitors can also promote Lmx1a activity. Panel B demonstrates that incubation with the DNA-PK inhibitor for PA6-induced progenitors also upregulated mRNA transcripts for Notch effectors; *Hes5* and *Hey1* and target gene *Pax6*. ***p<0.001, **p<0.01 and *p<0.05 one-way ANOVA with Bonferroni’s post-hoc test. Data expressed as mean ± SEM, n=3.

To determine whether the signaling mechanisms driving Lmx1a activity are protocol specific, we differentiated *Lmx1a*-*luc* mESCs by co-culture on PA6 stromal cells. PA6-induced neural progenitors were incubated with a selection of SMAs between days 2-6 or days 4-8 and analysed on day 6 or 8 (Figure **6A**), respectively. Analysis of luciferase activity revealed that incubation with DNA-PKi, PI3Ki (10 µM), DNA-PKi + EGFRi and EGFRi + VEGFRi from day 4-8 all significantly (p<0.05, one-way ANOVA post-hoc Bonferroni’s test) increased luciferase expression to around 180% of vehicle, Figure **6A**. Inhibitors of DNA-PK and PI3K signaling were the only compounds able to modulate luciferase activity alone. Real-time qPCR analysis for Notch effectors was performed on PA6-induced neural progenitors incubated with DNA-PKi. By day 8, a significant (p<0.05) upregulation in *Hes5*, *Hey1* and *Pax6* mRNA transcripts had occurred (1.53 ±0.09, 2.11 ± 0.06 and 1.75 ±0.08-fold respectively, when compared to vehicle) Figure **6B**.

### Small molecule agents promote expression of post-mitotic markers

**Figure 7:**
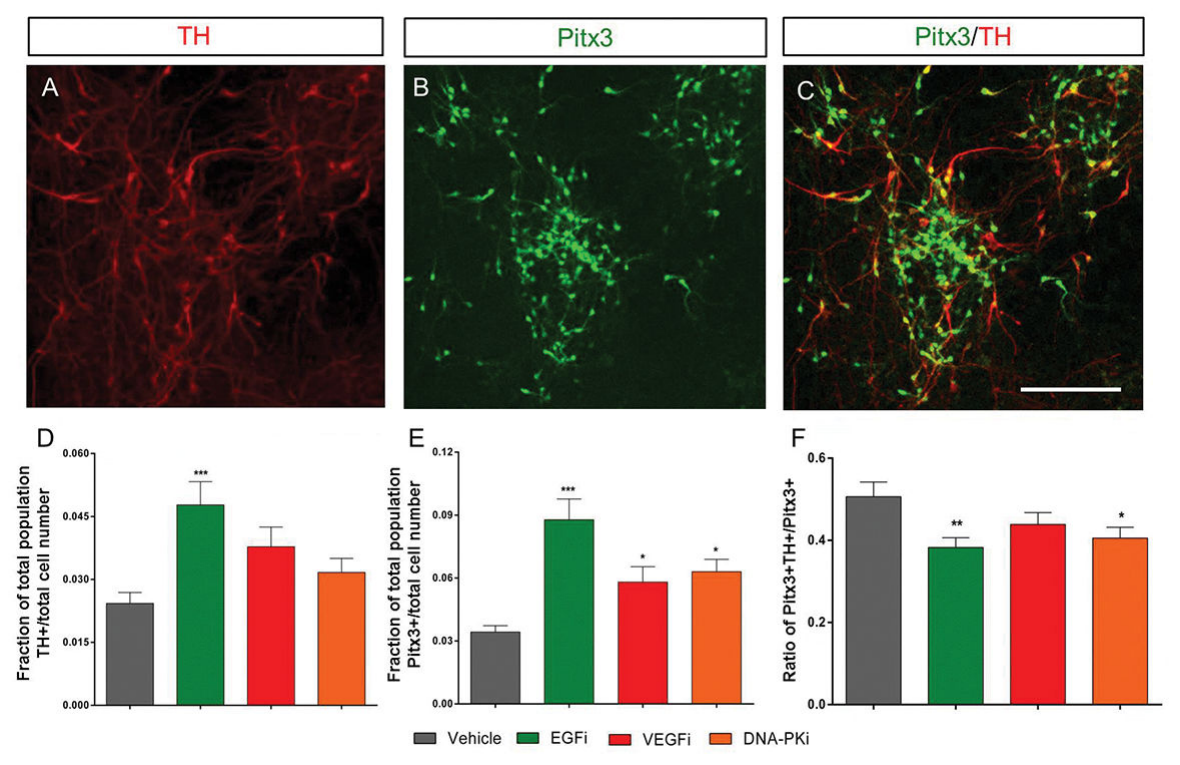
Small molecules can promote the specification of specific post-mitotic phenotypes. Neural progenitors incubated with SMAs gave rise to TH+ (A), Pitx3+ (B) and TH+/Pitx3+ neurons (C) by day 19 of differentiation. Pitx3 expression was determined using from residual GFP expression after fixation of cells produced by differentiating the *Pitx3*-*eGFP* mESC line, whereas visualization of TH was achieved using the secondary antibody anti-Rabbit AlexFluor594 binding to a Rabbit anti-TH primary antibody. Column bars show mean ± SEM fraction of Pitx3+ (D) and TH+ (E) cells present in each field of view, expressed as a fraction of the total cell counts (n = 35-36 fields of view from four replicate experiments). ***p<0.001 **p<0.01 and *p<0.05 respectively, using one-way ANOVA compared to vehicle with Dunnett’s multiple comparisons. Scale 100 µm.

Next we investigated the propensity for SMA-treated neural progenitors to develop into more mature dopaminergic neurons. Cultures of *Pitx3*-*eGFP* mESCs were exposed to SMAs from days 4-8 then further differentiated and assayed for expression of TH and Pitx3 on day 19 (Figure **7A-C**). Only incubation with the EGF inhibitor was able to significantly (one-way ANOVA, p<0.05) increase the yield of TH+ neurons in culture (1.8-fold increase over vehicle), Figure **7D**. Despite this, EGF, VEGF and DNA-PK inhibitors all significantly (p<0.05) increased the number of Pitx3+ neurons present by day 19 with 2.5-, 1.7- and 1.8-fold elevation over vehicle controls, respectively, Figure 7E. These data indicate that DNA-PK and VEGF inhibitors stimulate the expression of Pitx3 without a corresponding elevation of TH, resulting in an apparent decrease in the TH^+^+Pitx3^+^ / Pitx3^+^ ratio, Figure **7F**. 

## Discussion

The complex interplay between signaling pathways that drive neural differentiation makes specific mechanisms difficult to identify. Using SMAs to selectively activate or inhibit signaling pathways, coupled with genetic reporters to track neural differentiation [17] is a strategy that has the potential to identify particular signaling pathways to allow acquisition of specific subtypes of differentiating ESCs [23,24]. The increasing awareness and understanding that small molecules can induce ESC differentiation, is resulting in more published differentiation protocols that use SMAs rather than polypeptide growth factors [25]. Recombinant proteins such as sonic hedgehog (Shh) and Wingless (Wnt) proteins are important directors of development, but are expensive and show inconsistent activities. Thus, SMAs may be cheaper and more reliable; in recent studies they have, for example, been used to induce dopaminergic development by promoting Shh and Wnt signaling [5,26,27]. 

In this study we used mESC reporter lines that enabled the identification of Lmx1a+ progenitor cells as well as post-mitotic midbrain neurons. Lmx1a is a necessary signaling component of dopaminergic specification and development [1] with numerous signaling pathways contributing to the generation of Lmx1a+ mDA progenitors [6,12,28]. We initially identified 16 SMAs involved in controlling Lmx1a activity. These SMA’s implicated; EGF, VEGF, DNA-PK, PKC, NF-κB and Cyclin dependent kinase (CDK) pathways as regulators of Lmx1a, and therefore, luciferase activity (Figure **1**) without major changes in cell viability, Figure **S1**. Tyrosine kinase signaling is widespread in neuronal development, with EGF, FGF and VEGF receptors signaling known to be important in promoting dopaminergic differentiation [29,30], although the downstream signals responsible are not fully characterized. Our reporter screens identified multiple SMAs that induced Lmx1a activity and the midbrain neuronal phenotype through modulation of EGFR, VEGFR (Figure **7**) and DNA-PK signaling pathways, Figures **5** and **7**.

Curiously, the inhibition of PI3K signaling did not reverse the increase in Lmx1a activity seen following VEGF inhibition (Figure **2**) indicating that, in the absence of VEGF signaling, PI3K is not essential for driving Lmx1a activity. In contrast, the addition of the DNA-PK inhibitor totally abolished the increase in Lmx1a activity (Figure **2**), suggesting that when VEGF signaling is restricted, DNA-PK is critical for the expression of Lmx1a. During active EGF signaling, DNA-PK associates with, and is phosphorylated, by the EGF receptor [31]. Therefore, we propose that, in the absence of VEGF signaling, EGF, or other RTK signaling drives DNA-PK, but not PI3K, to elevate Lmx1a. The finding that incubation with the EGF inhibitor permitted a modest increase in Lmx1a (Figure **2**) would indicate that, following a reduction of EGF signaling, endogenous signaling mechanisms are still capable of driving Lmx1a signaling that is independent of both PI3K and DNA-PK signaling. Together these outcomes suggest that the absence of VEGF signaling strongly drives Lmx1a induction through a DNA-PK, but not PI3K pathway. Moreover, in the absence of EGF signaling, a mechanism capable of weakly driving Lmx1a activity is present and is independent of PI3K and DNA-PK, Figure **2**. 

DNA-PK belongs to the atypical protein kinase family of phosphatidylinositol 3-kinase-related kinases (PIKK) [32] and has a central role in the repair of DNA damage through non-homologous end joining (NHEJ) repair mechanisms [33]. Other PIKK family members such as ataxia telangiectasia mutated (ATM) and ATM and RAD3-related (ATR) have critical roles in single [34] and double-stranded [35] DNA damage repair. Due to a conserved kinase domain, it was initially thought that DNA-PK could act as a lipid kinase to activate PI3K targets [36] and has recently been shown to play additional roles in maintaining cell viability by directly phosphorylating Akt at Ser-473 in a stimulus specific manner [37]. In addition, aside from the PI3K pathway [32], other targets of DNA-PK inhibition may include regulation of the cell cycle [38]. Therefore, in the absence of VEGF, it is possible that DNA-PK inhibition may regulate several targets in neural progenitors to induce differentiation.

To further characterize the downstream signaling pathways contributing to Lmx1a expression, a number of highly selective signaling pathway inhibitors were used. The MAPK pathway is critical in maintaining ESC pluripotency [39] and is an important contributor to the dopaminergic specification of neuroectoderm [40,41]. The dramatic decrease in Lmx1a in response to MEK inhibition (Figure **3**) is entirely consistent with reports that ERK1/2 signaling is required to promote lineage specification [21,42]. Notably, although MEK inhibition decreased Lmx1a activity, it did not cause cell death, Figure **S2**. These data indicate that not only is our model system valid, but that there is an endogenous activator of MEK/ERK signaling that promotes dopaminergic neurogenesis, possibly via Raf. In contrast, the lack of effect of the p38-MAPK and JNK inhibitors indicates that in the presence of the endogenous signaling these pathways may not be necessary for midbrain specification (Figure **3**).

Phospholipase C-gamma (PLC-γ) is another signal transduction system that is activated by a number of RTKs [43]; inhibition of this pathway decreased Lmx1a reporter gene expression, implicating PLC-γ as a modulator of Lmx1a. Our early data showed that the inhibition of PKC, one of the downstream effectors of PLC-γ signaling, was detrimental to Lmx1a in developing cultures, Figure **1**. The inhibition of PKC did not kill cells in culture (Figure **S1**), but nor did it increase Lmx1a activity (Figure **1** and **3**). This data leads us to believe that RTKs activate PLC, resulting in the stimulation of the MEK/ERK pathway (via Ras-Raf) without concomitant activation of PKC. 

In contrast to our earlier finding where DNA-PK inhibition completely blocked the expression of Lmx1a induced by the suppression of VEGF signaling (Figure **2**), inhibition of DNA-PK signaling in the presence of intact VEGF and EGF signaling pathways directly elevated Lmx1a activity (Figure **3** and **4**). Given that the direct interaction between the EGFR and DNA-PK is well established [31], we believe that this system drives Lmx1a, however, it is also evident from these findings that this activity is entirely context dependent. We would speculate that, in the presence of intact VEGF and EGF signaling pathways the cellular context has changed, favouring proliferation, rather than commitment and, under these conditions, suppression of DNA-PK signaling leads to Lmx1a expression. Similarly, in the presence of VEGF and EGF signaling, the inhibition of PI3K pushes cells toward an Lmx1a+ fate (Figure **3**). Moreover, since the inhibition of Akt was without effect on Lmx1a activity (Figure **3**) we believe that the effect of PI3K is independent of Akt. There are reports that signal crosstalk downstream of VEGF and EGF receptors occurs between PI3K [21,44] and Notch [45] within multiple tissue systems [46–48] and can occur both dependent and independent of Akt [49]. As this is the first report of DNA-PK involvement in neural specification, the neurogenic potential of cultures following the inhibition of DNA-PK was also investigated. The suggestion that Notch signaling may serve as an underlying mechanism for promoting generation of Lmx1a+ progenitors, is an important consideration for the proper use of monolayer differentiation, as Notch signaling in neural progenitors is highly context-dependent and generates multiple cell types [50]. 

Several reports illustrate that during neuroectodermal differentiation of ESCs, Notch signaling controls the proportion of neurogenic cell types such as neural stem cells (NSCs) through direct repression of bHLH proneural factors by *Hes* and *Hey* genes [51,52]. Our hypothesis that specific inhibition of DNA-PK may increase neurogenic potential of ectodermal cultures is consistent with our data showing an upregulation of *Pax6* and *Hes5* in response to the inhibition of DNA-PK (Figure **5**). These genes are essential for the maintenance of Lmx1a+ neuroepithelial and radial glia (RG) cell types [53]. The upregulation of mRNA transcripts for midbrain (*En1*), but not the forebrain *FoxG1*, in response to DNA-PK inhibition (Figure **5**) may signify a developmental tendency towards midbrain specification. In the developing mouse, apart from RG cells, *Pax6* expression demarcates cells of the dorso-caudal forebrain and actively represses *En1*, establishing a diametrically opposed expression domain in the midbrain [54]. Therefore, it is possible that Lmx1a+ cells expressing *Pax6* or *En1* reflect separate dorsal populations. However, given that the transcription of forebrain specific mRNA was unaffected by inhibition of DNA-PK, we believe that DNA-PKi may favour the generation of RG-like cell types that are responsive to signals that promote regional specification but not subtype specification, as ventral dopaminergic markers Corin and FoxA2 [55,56] were down regulated in presence of the DNA-PKi, Figure **5**.

The difficulty in characterizing small molecule regulation of a PI3K/Notch underlying pathway is linked to context-specific nature of this mechanism. To determine whether these pathways were also protocol specific, a co-culture protocol using PA6 stromal cells was investigated. The observation that DNA-PKi, PI3Ki and combinations of EGFi + VEGFi and DNA-PKi + EGFi could regulate Lmx1a activity during PA6 differentiation (Figure **6**) is similar to monolayer, but not identical, given that inhibitors of DNA-PK and PI3K were the only inhibitors to regulate Lmx1a alone, Figure **6**. Despite this, the upregulation of mRNA transcripts for Notch signaling in PA6-induced ESCs incubated with DNA-PKi (Figure **6**) paralleled the transcriptional profile in monolayer cultures (Figure **5**) and supports the notion that DNA-PK signaling contributes to an underlying signaling mechanism regulating Lmx1a activity.

Quantifying neural subtypes using co-culture methods are difficult as cell clusters can obscure individual cells, therefore, to quantitate the generation of neural subtypes the monolayer protocol was revisited. Quantitative FACS analysis confirmed that the generation of Lmx1a+ cells in the presence of the DNA-PK inhibitor requires active PI3K signaling, Figure **4**. Although inhibition of DNA-PK clearly regulates Lmx1a+ cell numbers, the downstream mechanisms activated in response to DNA-PKi are diverse. The data in Figure **4** show that mTOR complex 1 (mTORC1) signaling is essential in generating Lmx1a+ cells and that Lmx1a+ cell numbers are restored with co-incubation with the DNA-PK inhibitor. Moreover, incubation with the DNA-PK inhibitor was able to regulate activation of mTORC1 through phosphorylation at Ser2448, Figure **3**. Consistent with the changes in Lmx1a, the inhibition of EGF, VEGF or DNA-PK signaling also increased the yield of Pitx3+ neurons, Figure **7**. However, this increase in Pitx3+ neurons was not, with the exception of cultures generated in the absence of EGF signaling (Figure **7**), consistent with the expression of TH. If indeed DNA-PK activity is regulated by intact EGF signaling [57,58] and DNA-PK inhibition, can promote a more neurogenic cell type, EGF inhibition may control a switch toward differentiation and enhance the generation of mature neural subtypes. 

At this stage it is unclear if the enhanced expression of the TH-/ Pitx3+ cells represents a phenotype other than the midbrain dopaminergic neurons. Recent generation of a *Pitx3*-*Cre* knock-in mouse identified several regions in the midbrain that yield Pitx3+/TH- subtypes [59]. The rostral linear nucleus of the raphe (RLi) region contains Pitx3+/TH- neurons that develop more dorsal-medially to the mDA-rich zones of substantia nigra and the ventral tegmentum area. However, due to the omission of patterning factors in our study, it is likely that neural progenitors either; do not properly ventralize to a mDA fate (Pitx3+/TH+) or these Pitx3+/TH- neurons are yet to mature as TH+ dopaminergic neurons, since Pitx3 is known to induce expression of TH [60]. 

## Conclusions

In this study we used a reporter under the control of the *Lmx1a* promoter to screen a small molecule kinase inhibitor library and identify signaling pathways that modulate Lmx1a activity. Lmx1a activity was increased by suppression of EGF, VEGF and DNA-PK signaling pathways. Of these pathways, DNA-PK has never been identified as a regulator of dopaminergic differentiation. Further investigations showed that DNA-PKi upregulated Notch signaling and correlated with the appearance of regionalization markers in Lmx1a+ cells, promoting midbrain specification. Moreover, the upregulation regionalization markers using monolayer and co-culture paradigms may indicate a conserved mechanism for DNA-PK signaling. This increase in Lmx1a expression, while consistent with an increase in the midbrain specific Pitx3, does not, however, correlate with an increase in TH expression, indicating that additional patterning factors may be required to complete mDA specification. 

## Supporting Information

Figure S1 **Viability of cells incubated with small molecule agents.** Incubation of cultures with CellTiter Blue (CTB) showed that the 16 small molecule kinase inhibitors studies in further detail were able to modulate Lmx1a activity during the initial screens with little effect on cellular viability.
(TIF)

Figure S2 **The effect of modulating intracellular signaling cascades on cellular viability.** The majority of cultures treated with SMAs showed no difference in cell viability. However, a significant increase in cell growth was observed in response to MEK inhibition, PD and inhibition of PTEN in combination with Akt inhibition whereas incubation with Rapamycin significantly decreased cell viability. Data expressed as mean ± SEM of at least three independent experiments. ***p<0.001 and *p<0.05, respectively when compared to vehicle using one-way ANOVA with Bonferroni’s post-test.
(TIF)

Figure S3 ***Lmx1a*-*Luc* and *Lmx1a*-*AMP* constructs.** Panel A shows vector maps of constructs used to target exon 1 of Lmx1a in E14Tg2a ESCs. Constructs were introduced into ESCs by electroporation using GenePulser™ XCell electroporator (Bio-Rad Laboratories, USA). G418 was used for positive selection to determine successful integration of neomycin-containing construct. Panel B shows a cartoon of the targeting strategy for vectors to the *Lmx1a* *locus*. To confirm successful integration to the *Lmx1a* locus, genomic DNA from colonies was initially screened by PCR using primers for genomic *Lmx1a*. Successful clones of *Lmx1a-luc-IRES-eGFP* were subsequently screened by Southern blotting after DNA digestion with restriction enzyme AflII (Panel C) and hybridization of probes complementary to the *Lmx1a* sequence upstream of the 5’ homology arm of the vector and downstream of the 3’ homology arm. No clones tested showed extra bands suggesting single integrations. Given the intense bands observed for both 5’ and 3’ probes of clone 38 (red box), this *Lmx1a* clone was used for all luciferase screening assays.
(TIF)

Table S1 **Table of primary and secondary antibodies used.** All concentrations of antibodies used were empirically determined before use and stored according to manufacturer’s instructions.
(TIF)

Table S2 **Table of primer sequences used for qPCR.** Using the following protocol, 3 ng of cDNA, was used per reaction tube. Samples were heated initially at 95°C for 5 min, then 95°C; 10 seconds, 60°C; 30 seconds and 72°C; 30 seconds then repeated for 39 cycles. Key: GAPDH- Glyceraldehyde 3-phosphate dehydrogenase, *ACTB*- β-Actin, *En1*-Engrailed 1, *FoxG1*- Forkhead box protein G1, *FoxA2*- Forkhead box protein A2, *Hes5*- Hairy Enhancer of Split 5, *Hey1*- Hairy/Enhancer-of-split related with YRPW motif 1, *Pax6*- Paired box gene 6 and bp-base pairs.
(TIF)
