# Supplementary figures and images for: DNA-Dependent Protein Kinase Is a Context Dependent Regulator of Lmx1a and Midbrain Specification
Source: PLoS One. 2013 Oct 23;8(10):e78759. doi: 10.1371/journal.pone.0078759 (PMC3806860; doi:10.1371/journal.pone.0078759)

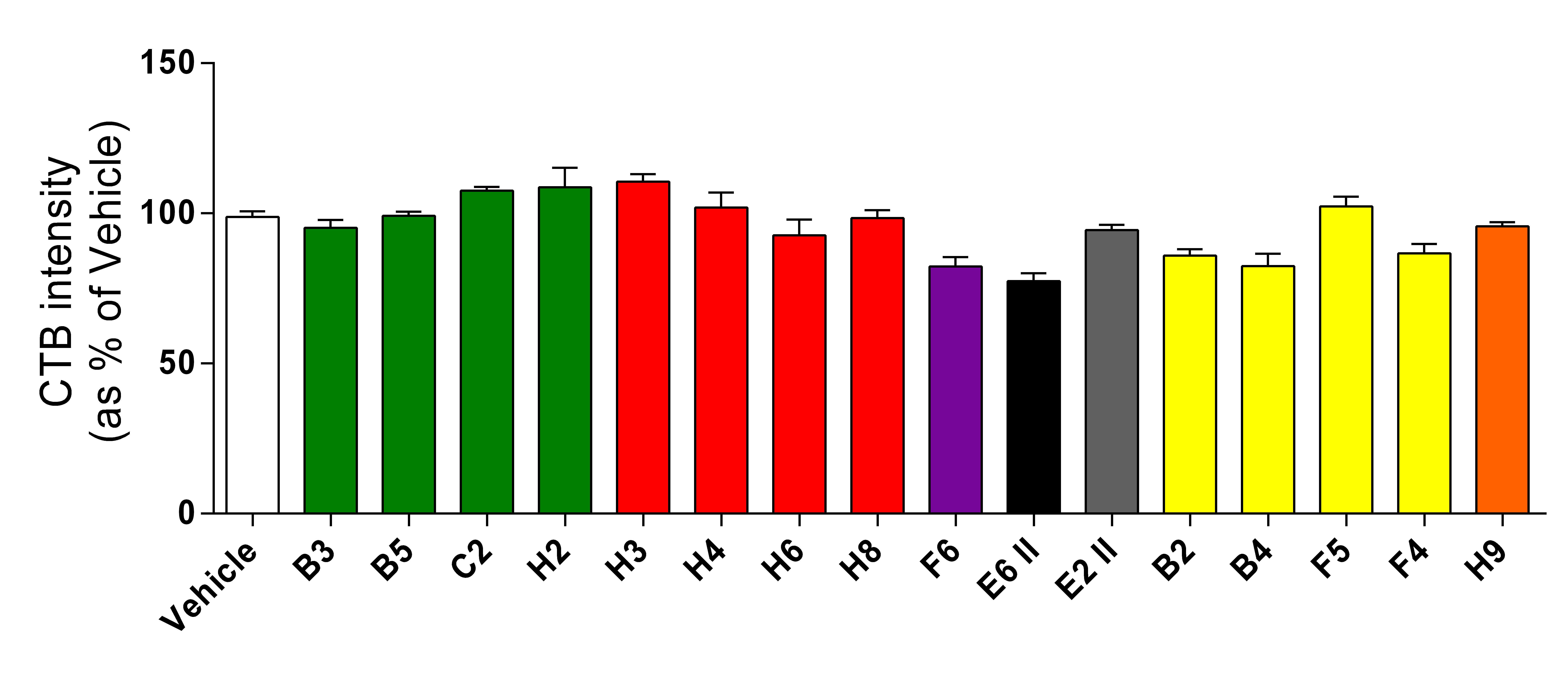

Supplement: Figure S1 — Viability of cells incubated with small molecule agents. Incubation of cultures with CellTiter Blue (CTB) showed that the 16 small molecule kinase inhibitors studies in further detail were able to modulate Lmx1a activity during the initial screens with little effect on cellular viability. (TIF) [file pone.0078759.s001.tif]

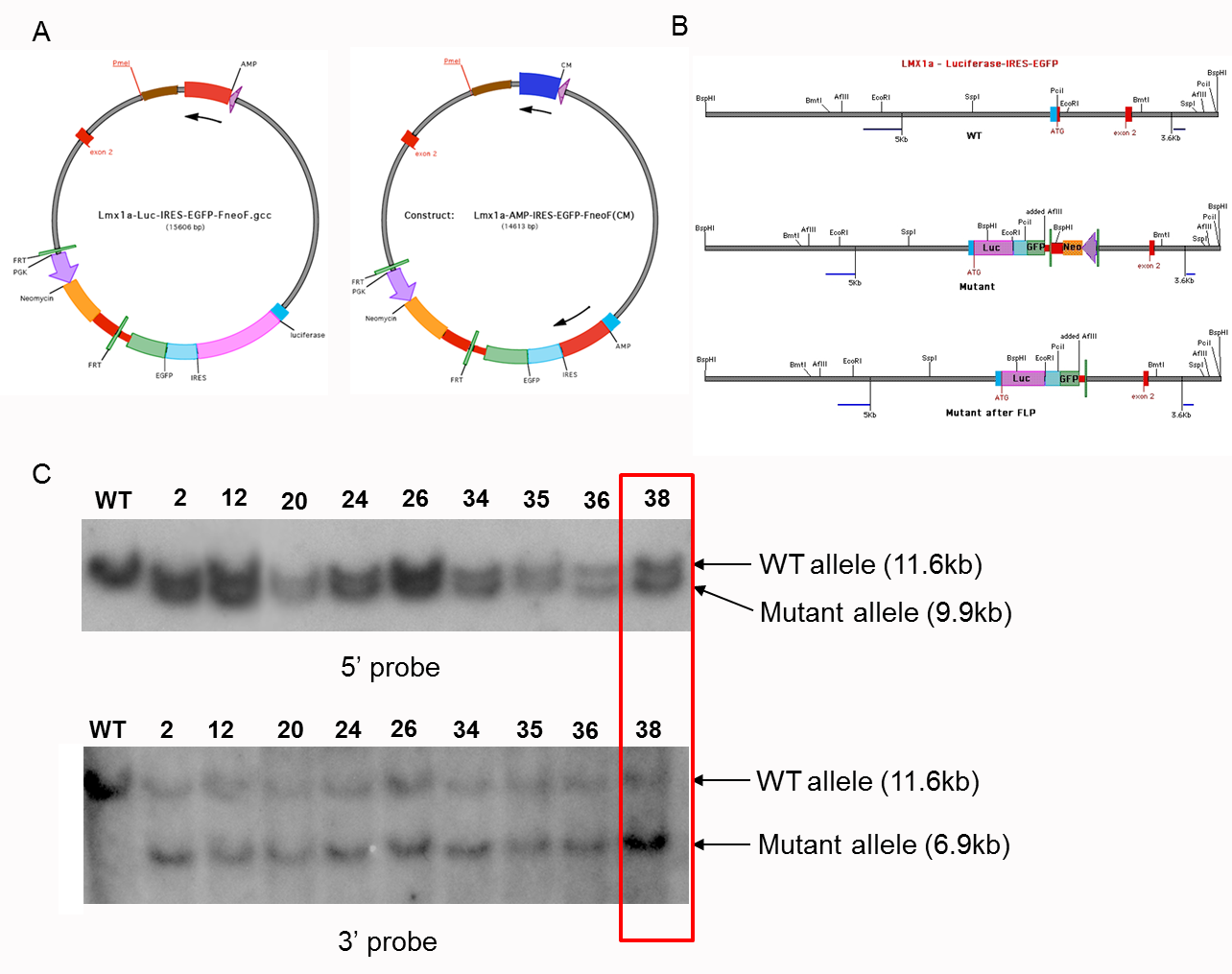

Supplement: Figure S3 — Lmx1a-Luc and Lmx1a-AMP constructs. Panel A shows vector maps of constructs used to target exon 1 of Lmx1a in E14Tg2a ESCs. Constructs were introduced into ESCs by electroporation using GenePulser™ XCell electroporator (Bio-Rad Laboratories, USA). G418 was used for positive selection to determine successful integration of neomycin-containing construct. Panel B shows a cartoon of the targeting strategy for vectors to the Lmx1a locus. To confirm successful integration to the Lmx1a locus, genomic DNA from colonies was initially screened by PCR using primers for genomic Lmx1a. Successful clones of Lmx1a-luc-IRES-eGFP were subsequently screened by Southern blotting after DNA digestion with restriction enzyme AflII (Panel C) and hybridization of probes complementary to the Lmx1a sequence upstream of the 5’ homology arm of the vector and downstream of the 3’ homology arm. No clones tested showed extra bands suggesting single integrations. Given the intense bands observed for both 5’ and 3’ probes of clone 38 (red box), this Lmx1a clone was used for all luciferase screening assays. (TIF) [file pone.0078759.s003.tif]

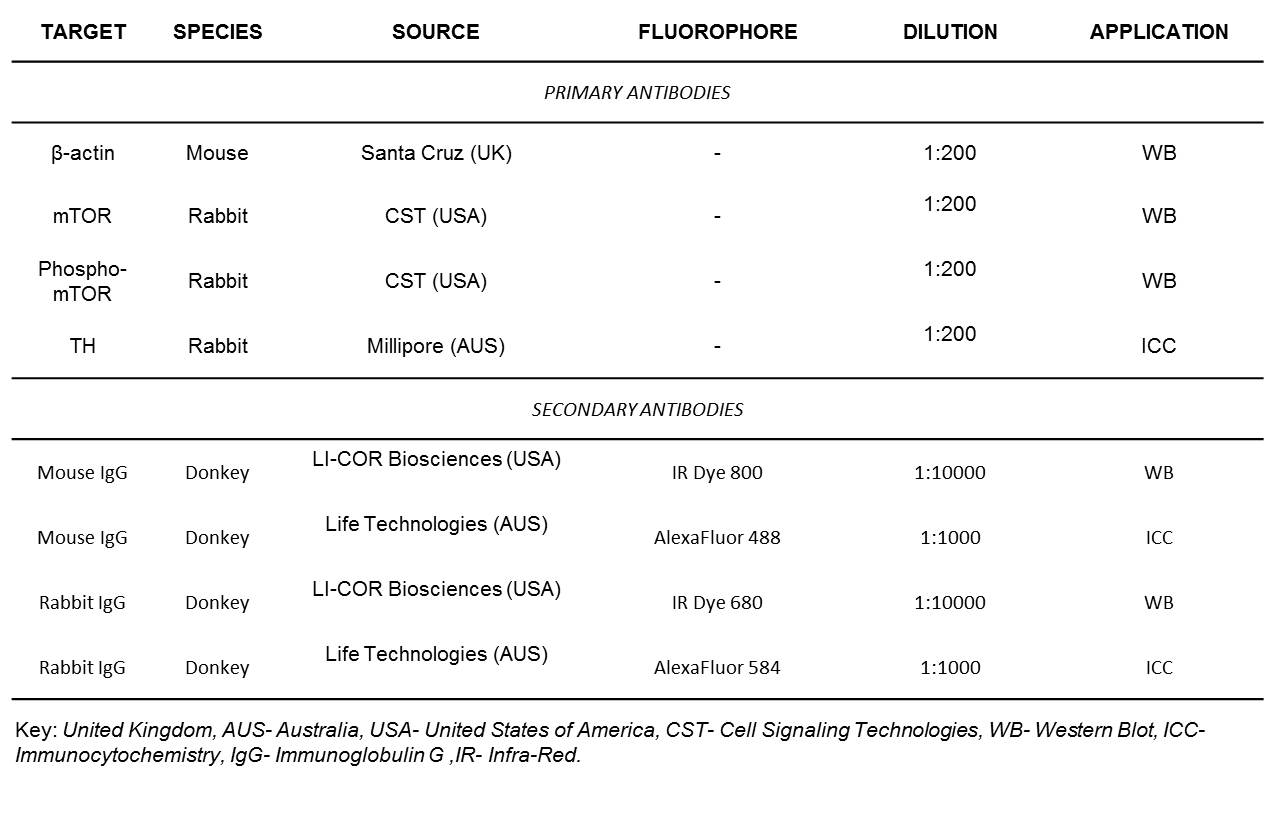

Supplement: Table S1 — Table of primary and secondary antibodies used. All concentrations of antibodies used were empirically determined before use and stored according to manufacturer’s instructions. (TIF) [file pone.0078759.s004.tif]

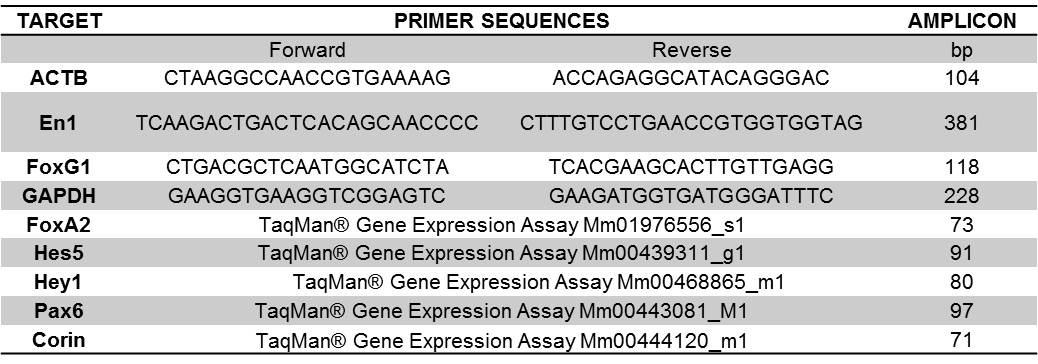

Supplement: Table S2 — Table of primer sequences used for qPCR. Using the following protocol, 3 ng of cDNA, was used per reaction tube. Samples were heated initially at 95°C for 5 min, then 95°C; 10 seconds, 60°C; 30 seconds and 72°C; 30 seconds then repeated for 39 cycles. Key: GAPDH- Glyceraldehyde 3-phosphate dehydrogenase, ACTB- β-Actin, En1-Engrailed 1, FoxG1- Forkhead box protein G1, FoxA2- Forkhead box protein A2, Hes5- Hairy Enhancer of Split 5, Hey1- Hairy/Enhancer-of-split related with YRPW motif 1, Pax6- Paired box gene 6 and bp-base pairs. (TIF) [file pone.0078759.s005.tif]
